# Death Certificates Underestimate Infections as Proximal Causes of Death in the U.S

**DOI:** 10.1371/journal.pone.0097714

**Published:** 2014-05-30

**Authors:** Sushant Govindan, Letitia Shapiro, Kenneth M. Langa, Theodore J. Iwashyna

**Affiliations:** 1 Department of Medicine, University of Michigan, Ann Arbor, Michigan, United States of America; 2 VA Center for Clinical Management Research, HSR&D Center for Excellence, Ann Arbor, Michigan, United States of America; 3 Institute for Social Research, Ann Arbor, Michigan, United States of America; Kyushu University Faculty of Medical Science, Japan

## Abstract

**Background:**

Death certificates are a primary data source for assessing the population burden of diseases; however, there are concerns regarding their accuracy. Diagnosis-Related Group (DRG) coding of a terminal hospitalization may provide an alternative view. We analyzed the rate and patterns of disagreement between death certificate data and hospital claims for patients who died during an inpatient hospitalization.

**Methods:**

We studied respondents from the Health and Retirement Study (a nationally representative sample of older Americans who had an inpatient death documented in the linked Medicare claims from 1993–2007). Causes of death abstracted from death certificates were aggregated to the standard National Center for Health Statistics List of 50 Rankable Causes of Death. Centers for Medicare and Medicaid Services (CMS)-DRGs were manually aggregated into a parallel classification. We then compared the two systems via 2×2, focusing on concordance. Our primary analysis was agreement between the two data sources, assessed with percentages and Cohen's kappa statistic.

**Results:**

2074 inpatient deaths were included in our analysis. 36.6% of death certificate cause-of-death codes agreed with the reason for the terminal hospitalization in the Medicare claims at the broad category level; when re-classifying DRGs without clear alignment as agreements, the concordance only increased to 61%. Overall Kappa was 0.21, or “fair.” Death certificates in this cohort redemonstrated the conventional top 3 causes of death as diseases of the heart, malignancy, and cerebrovascular disease. However, hospitalization claims data showed infections, diseases of the heart, and cerebrovascular disease as the most common diagnoses for the same terminal hospitalizations.

**Conclusion:**

There are significant differences between Medicare claims and death certificate data in assigning cause of death for inpatients. The importance of infections as proximal causes of death is underestimated by current death certificate-based strategies.

## Introduction

Conventional tabulations of death certificate data emphasize the underlying cause of death—usually chronic illness—but seem to have significant shortcomings with respect to both accuracy and methodology [1]–[3]. Furthermore, the current system may inadvertently lead to the impression that infectious etiologies are “solved problems” in the developed world, despite the efforts by some to contest this view [4], [5]. Indeed, recent Eurostat Cause of Death tabulations do not even include an infectious category in the most accessible presentation [6]. Nonetheless, infectious etiologies remain a major driver of hospitalization [7], and with over forty percent of deaths in the U.S. occurring during hospitalizations [8], it is plausible that the true importance of infections in hospital associated mortality is less apparent in conventional death certificate data.

Diagnoses of terminal hospitalizations may provide an alternative perspective to death certificates on cause of death, emphasizing the proximal causes towards which much current health expenditures are oriented [9], [10]. Claims for terminal hospitalizations include a diagnosis-related grouping (DRG) of the primary diagnosis that is well tracked and audited given that reimbursement is tied to DRGs. Additionally, the relative accuracy of DRGs has been substantiated by current literature [11], albeit with known limitations often more related to subtleties of complexity rather than the general type of disease. This provides a rationale of utilizing terminal hospitalization data in a complementary role for vital statistics reporting.

We therefore sought to test two hypotheses regarding patients who die as inpatients: (1) at the individual-level, there would be poor agreement between death certificate-derived causes of death and the proximal causes of death as revealed by the primary diagnosis of a terminal hospitalization; and (2) there would be systematic under-representation of infections in death certificate data relative to terminal hospitalization data. Unlike most past studies of the accuracy of death certificates, which often rely on data from only a single/few institutions or examine only one pathology [12]–[19], we examined all inpatient deaths across multiple pathologies in the Health and Retirement Study (HRS)—a nationwide sample of older Americans—who had linked Medicare claims and National Death Index (NDI) data from 1993–2007 (the latest years for which all data is available).

## Methods

Data were derived from the nationally-representative HRS study, an NIH-funded longitudinal cohort study that has been ongoing since 1992. Hospitalization data was from the linked HRS-Medicare files. All HRS respondents are followed in the National Death Index, as well. The University of Michigan Institutional Review Board approved this work. Patients provided informed consent on enrollment in the HRS and again for linkage to Medicare claims.

All deaths in the linked HRS-Medicare data were eligible for inclusion. Patients who die as an inpatient in principle should have a claim where the discharge date is set as their death date and should have the same date reflected on their death certificate; however, given known imprecisions in the timing on such paperwork, inpatient death was defined as follows: a death date on the death certificate within 1 day of hospital discharge date. The primary DRG was abstracted from such terminal hospitalizations. Patients were included if birth date, death date, and Medicare records were available, and they were successfully matched to death certificate data from the National Death Index. They were excluded if no DRGs or valid cause of death was coded. All patients were true matches by NDI classification criteria.

Causes of death abstracted from death certificates were aggregated to the standard National Center for Health Statistics List of 50 Rankable Causes of Death. DRGs were aggregated into a crosswalk/parallel classification by a physician blinded to the death certificate information in order to create a system that allowed for comparison of DRG data with death certificate data. We then compared the two systems via a cross-classification table, focusing on overall concordance and concordance amongst the top 3 causes of death. As some DRGs do not naturally align with a cause of death (e.g. DRG 75: Major Chest Procedures), we examined the sensitivity of our conclusions to reclassification of such “unaligned” DRGs. “Influenza and Pneumonia” and “Septicemia” were combined in our Tables, given modern understandings of severe sepsis, into “Infections”.

Our primary analysis was agreement between the two data sources, assessed with percentages and Cohen's kappa statistic, classified by Landis & Koch's 1977 criteria [20]. As a sensitivity test, we confirmed that these results were unchanged when considering differences between ICD-9 and ICD-10-coded death certificates. Stata 12.1 was used to perform the analysis.

## Results

Overall, 2074 inpatient deaths were included in our analysis; the demographics for the cohort can be seen in Table 1. Mean age at death was 80.4, and 52.2% were female.

**Table 1:** Demographics of Study Cohort (N = 2074).

AGE (mean)	80.4
Men, # (%)	991 (47.8)
**Year of Death, # (%)**	
1993	2 (.1)
1994	125 (6.0)
1995	130 (6.3)
1996	156 (7.5)
1997	150 (7.2)
1998	144 (6.9)
1999	157 (7.6)
2000	176 (8.5)
2001	178 (8.6)
2002	178 (8.6)
2003	170 (8.2)
2004	161 (7.7)
2005	151 (7.3)
2006	128 (6.2)
2007	68 (3.3)
**ICD Classification, # (%)**	
9	707 (34.1)
10	1367 (65.9)

Only 36.6% of death certificate cause-of-death codes agreed with the reason for the terminal hospitalization in the Medicare claims at the broad category level. When re-classifying DRGs without clear alignment as agreements, the concordance only increased to 61%. Overall Kappa was 0.21 (S.E. 0.01), “fair agreement”. Kappa for ICD 9 and ICD 10 based death certificates were 0.30 (S.E. 0.01) and 0.24 (S.E. 0.01), respectively.

Additionally, 41.0% of all patients in our cohort with a death certificate COD of heart disease, malignancy, or cerebrovascular disease had a consonant DRG diagnosis during their terminal hospitalization (68.3% when considering unaligned DRGs as agreement). At least 1 in 6 deaths from heart disease, malignancy or cerebrovascular disease occurred during a hospitalization for influenza, pneumonia or septicemia (Table 2).

**Table 2:** Comparison of Cause of Death Agreement between DRGs and Death Certificates for Common Causes of Death.

		Medicare Claim/Primary Diagnosis Category from Inpatient Hospitalization During Which Patient Died, # (row %)		
		*Agreement, Unaligned DRGs Considered Disagreement*	*Agreement, Unaligned DRGs Considered Agreement*	*Infections*
**Death Certificate Derived (# of Patients for Each Category)**	*Diseases of the Heart (639)*	213 (33%)	421 (66%)	123 (19%)
	*Malignancy (319)*	120 (38%)	217 (68%)	54 (17%)
	*Cerebrovascular Disease (195)*	105 (54%)	138 (71%)	37 (19%)

Use of DRGs rather than death certificates resulted in a significant reordering of causes of death for inpatients. Death certificate data rank the top 3 causes of death as heart disease, malignancy, and cerebrovascular disease (Table 3), whereas DRG data rank infections, diseases of the heart, and cerebrovascular disease as leading etiologies.

**Table 3:** Top 3 Causes of Death: National Vital Statistics 2007 vs Death Certificates vs DRG.

	Source of Data		
	National Vital Statistics	Death Certificates from HRS-Medicare Inpatient Decedents	DRGs from HRS-Medicare Inpatient Decedents
**Cause of Death Categories in Order of Frequency**	1. Diseases of the Heart (25.4%)	1. Diseases of the Heart (30.8%)	1. Infections (24.5%)
	2. Malignancy (23.2%)	2. Malignancy (15.4%)	2. Diseases of the Heart (13.5%)
	3. Cerebrovascular Disease (5.6%)	3. Cerebrovascular Disease (9.4%)	3. Cerebrovascular Disease (7.2%)

## Discussion

Our study demonstrates that death certificates and hospital records paint distinct and complementary impressions about mortality. Death certificate data currently emphasizes the burdens of chronic disease. However, a clear majority of deaths occur during hospitalizations for reasons quite different from the reported underlying cause of death. This suggests that national mortality data often understate the proximal cause of death during that terminal hospitalization; specifically, there appears to be a neglect of the proximal role of infections and sepsis, particularly in older adults. From 17 to 19% of inpatient deaths attributed to heart disease, cancer and stroke by death certificates nonetheless occur during hospitalizations for infections. A balanced approach to harm mitigation for these chronic diseases still involves attention to and advances in the care of acute infections.

The current approach to death certificate data—with its disaggregation of infectious causes leading to infections not being in the top 5 causes of death as listed by the CDC [21]—may reflect a false view that, in the developed world, infectious diseases are a solved problem, save perhaps for newly emerging or highly resistant infections. Indeed, the results of this study bring to the forefront the apparently contradictory findings that infections are a major cause for hospitalizations in the U.S.[7] and yet none of the top 7 causes of death nationally are of an infectious nature [22]. When considering the entity of sepsis, current trends seem to be at odds, as well; multiple studies on sepsis show significant morbidity and mortality [23]–[28], increasing incidence [29], and it being the most expensive etiology of acute hospitalization in the United States [7]. Just as good diabetes care includes good cardiovascular and renal care, our data raise the hope that meaningful opportunities to improve cardiovascular and oncologic mortality may lie in the more effective and better implemented treatment of infections and severe sepsis [30]. Thus recent working on emerging and re-emerging infections may be appropriately viewed as of central importance even in the developed world [31].

There are limitations with our study. First, the variability between the death certificate and DRG classification systems led to “unaligned” DRGs in our crosswalk, which did affect the precision of our results. However, our substantive conclusion of relatively poor agreement held even in the context of the most generous assessment of “unaligned” DRGs. Second, the crosswalk employed in this study was uniquely created during the project by the authors; therefore, it would need to be reproduced and verified in future studies, though our conclusions are not without support from current literature as cited above. Third, the population of our study includes only US hospitalized patients over the age of 51 – and predominantly aged 65 and above—which limits the generalizability of our conclusions. However, considering that at least 70% of deaths in the United States occur in this 65+ age group [22], that over forty percent of deaths occur in the hospital in the U.S., and that a large proportion of healthcare expenditures are oriented towards hospitalized patients, the implications of this study on U.S. healthcare remain of policy importance. Fourth, there are concerns in the literature regarding administrative data, its inherent biases, and methodological concerns [32]–[35]; however, many experts would agree that administrative data has a place in healthcare statistics. Moreover, we are asserting that DRGs are a complimentary source of information, not a replacement for death certificates.

We believe our study demonstrates an incomplete understanding of mortality in the data often employed in the United States and likely elsewhere in the developed world. In the established system, proximal, infectious etiologies are underrepresented and underappreciated with respect to their impact on the healthcare of Americans. These proximal, infectious causes of death are potentially treatable [30], suggesting that a balanced portfolio of research and treatment might include more attention to them. In fact, this expanded role of infectious disease in causing death among those with severe heart disease has been recognized in recent calls for cardiologists staffing Coronary Care Units to increase their expertise in the treatment of severe sepsis and ARDS [36]. Equivalent changes may be needed in our reporting systems as they drive other aspects of public health preparation and care. The “short-term memory loss” of our existing mortality assessment system significantly limits the current understanding of what kills Americans in the 21^st^ century, potentially leaving opportunities for more efficient allocation of healthcare resources.
